# An active form of Vav1 induces migration of mammary epithelial cells by stimulating secretion of an epidermal growth factor receptor ligand

**DOI:** 10.1186/1478-811X-4-5

**Published:** 2006-05-18

**Authors:** Julie L Wilsbacher, Sheri L Moores, Joan S Brugge

**Affiliations:** 1 Department of Cell Biology, Harvard Medical School, 240 Longwood Avenue, Boston, MA 02115, USA; 2 Current address : Cancer Research, Global Pharmaceutical Research and Development, Abbott Laboratories, Abbott Park, Illinois 60064, USA; 3 Current address : GlaxoSmithKline, Oncology, Collegeville, PA 19426, USA

## Abstract

**Background:**

Vav proteins are guanine nucleotide exchange factors (GEF) for Rho family GTPases and are activated following engagement of membrane receptors. Overexpression of Vav proteins enhances lamellipodium and ruffle formation, migration, and cell spreading, and augments activation of many downstream signaling proteins like Rac, ERK and Akt. Vav proteins are composed of multiple structural domains that mediate their GEF function and binding interactions with many cellular proteins. In this report we examine the mechanisms responsible for stimulation of cell migration by an activated variant of Vav1 and identify the domains of Vav1 required for this activity.

**Results:**

We found that expression of an active form of Vav1, Vav1Y3F, in MCF-10A mammary epithelial cells increases cell migration in the absence or presence of EGF. Vav1Y3F was also able to drive Rac1 activation and PAK and ERK phosphorylation in MCF-10A cells in the absence of EGF stimulation. Mutations in the Dbl homology, pleckstrin homology, or cysteine-rich domains of Vav1Y3F abolished Rac1 or ERK activation in the absence of EGF and blocked the migration-promoting activity of Vav1Y3F. In contrast, mutations in the SH2 and C-SH3 domains did not affect Rac activation by Vav1Y3F, but reduced the ability of Vav1Y3F to induce EGF-independent migration and constitutive ERK phosphorylation. EGF-independent migration of MCF-10A cells expressing Vav1Y3F was abolished by treatment of cells with an antibody that prevents ligand binding to the EGF receptor. In addition, conditioned media collected from Vav1Y3F expressing cells stimulated migration of parental MCF-10A cells. Lastly, treatment of cells with the EGF receptor inhibitory antibody blocked the Vav1Y3F-induced, EGF-independent stimulation of ERK phosphorylation, but had no effect on Rac1 activation or PAK phosphorylation.

**Conclusion:**

Our results indicate that increased migration of active Vav1 expressing cells is dependent on Vav1 GEF activity and secretion of an EGF receptor ligand. In addition, activation of ERK downstream of Vav1 is dependent on autocrine EGF receptor stimulation while active Vav1 can stimulate Rac1 and PAK activation independent of ligand binding to the EGF receptor. Thus, stimulation of migration by activated Vav1 involves both EGF receptor-dependent and independent activities induced through the Rho GEF domain of Vav1.

## Background

The Rho family guanine nucleotide exchange factor (GEF), Vav1, plays a central role in transducing signals from cell surface receptors, such as integrin, growth factor and immune response receptors, to stimulate multiple cellular activities. These activities include many that involve changes in the actin cytoskeleton, such as lamellipodium and ruffle formation and cell spreading [1,2]. Vav1 expression is normally restricted to hematopoietic cells while its isoforms, Vav2 and Vav3, are more widely expressed [3-6]. All three Vav isoforms have been shown to be abnormally expressed in several types of cancer. Vav1 is ectopically expressed and is believed to have a role in increased cell proliferation and metastasis of pancreatic cancer cells [7,8], and it is also expressed in a subset of neuroblastomas [9]. In addition, based on SAGE data, Vav2 expression levels are increased in several types of brain cancers and Vav3 is overexpressed in breast carcinomas [10]. Vav1 overexpression enhances the activation of multiple intracellular signaling pathways including extracellular signal-regulated kinase (ERK), Jun N-terminal kinase (JNK), and phosphoinositide-3-kinase(PI3K)[1,11]. Vav proteins are composed of multiple domains that mediate protein interactions as well as catalytic activity [1,12-14]. By interacting with structural and signaling proteins, Vav1 may serve to integrate signals required to properly execute activation of downstream pathways. Thus, it is important to understand the mechanisms whereby Vav1 serves as a scaffold to coordinate with Rho family GTPases and other signaling and structural proteins to regulate changes in the actin cytoskeleton and activate intracellular signaling pathways.

Vav1, Vav2, and Vav3 are composed of multiple domains in addition to the Dbl homology (DH) domain that mediates Rho family GTP exchange. These domains include a calponin homology (CH) domain, a domain rich in acidic amino acids, a pleckstrin homology (PH) domain, a cysteine-rich (CR) domain, two Src homology (SH) 3 domains, and an SH2 domain [1,2,12-14]. The activities of several Vav domain mutants have been tested in vitro or in lymphoid cells or fibroblasts [6,15-23]. Deletion of the CH domain produces an active form of Vav, thus it has been proposed that this domain acts as a negative regulator of Vav, possibly through intramolecular binding to the cysteine rich domain [17,20,24,25]. However, the CH domain also has a role in activation of NFAT downstream of Vav1 in T cells, because deletion or mutation of this domain in Vav1 suppresses its activation of NFAT [14,18,19,26]. Within the acidic domain are three tyrosine residues that participate in an autoinhibitory interaction with the DH domain, thus blocking access of Rho GTPases [27]. The PH domain was hypothesized to regulate DH domain function by binding to PIP3 [1,28-30], but recent data suggest that phospholipids do not regulate activation of Vav [20,25,31]. However, the PH domain does seem to be required for Vav activity in cells by an unknown mechanism [20-22]. Mutation of the cysteine rich region of Vav1 blocks its ability to catalyze exchange of nucleotides on Rac or activate JNK in fibroblasts and Jurkat T cells, suggesting that this domain is required for catalytic activity [6,20,21,32]. The SH3-SH2-SH3 domains, collectively known as the adaptor region, have been shown to interact with several signaling proteins [1,12,14,33-40]. The requirement of each domain for signaling downstream from Vav in response to growth factor receptor or integrin activation *in vivo *has not been defined.

The adaptor region of Vav1 binds to many different proteins. The C-terminal SH3 (C-SH3) domain binds to several polyproline-containing proteins, including cytoskeletal proteins and RNA-binding proteins [14,33,35,40]. The Vav1 SH2 domain mediates binding of Vav to phosphotyrosine residues of growth factor receptors, kinases, phosphatases, and the SLP-76 adaptor protein [14,34,36-38]. All three Vav isoforms are phosphorylated on tyrosines following treatment of cells with several distinct growth factors and the tyrosine phosphorylation sites themselves serve as binding sites for other SH2 domain containing proteins [14,41-44]. Although the sequence of the N-SH3 ligand-binding region diverges significantly from the SH3 consensus and, to date, no polyproline ligands have been identified for this domain [1], it does bind to SH3 domains of the adaptor proteins Grb2 and Crk [45-47]. Thus, the Vav N-SH3 domain possesses the unique ability to interact with other SH3 domains. Vav is the only DH containing protein that contains an SH2 domain [1,48]. The presence of the SH2 and SH3 domains may allow Vav to couple with receptors as well as serve as a scaffold protein to recruit proteins required for its downstream signaling.

We have characterized the phenotypic effects of overexpression of an active form of Vav1, Vav1Y3F, in the human mammary epithelial cell line, MCF-10A. We show that Vav1Y3F causes morphological changes and increased migration of MCF-10A cells. Cells expressing Vav1Y3F also exhibit increases in Rac1, Pak, and ERK activation in the absence of growth factor stimulation. All these activities are dependent on the GTPase exchange activity of Vav1. However, the Vav1-induced increase in migration and ERK activation, but not activation of Rac1 and Pak, are dependent on the secretion of an epidermal growth factor (EGF) receptor ligand stimulated by Vav1Y3F. Thus, in MCF-10A cells, Vav1 activates migration and the ERK pathway indirectly through secretion of an EGF receptor ligand.

## Results and discussion

### Expression of active Vav1 in MCF-10A cells causes morphological changes and stimulates migration

**Figure 1:**
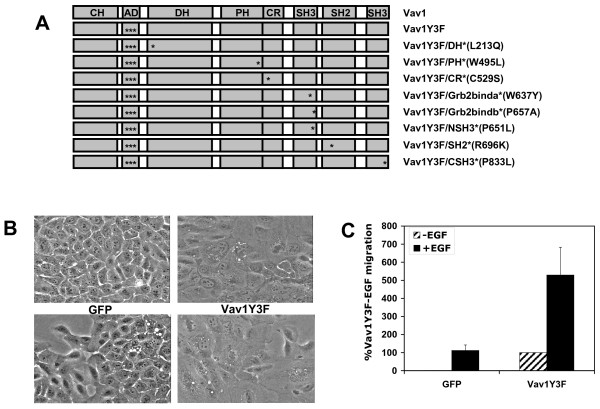
**Vav1Y3F stimulates morphological changes and increases migration of MCF-10A cells**. (A) Schematic illustration of mutations made in different domains of Vav1. The domain structure of Vav1 is illustrated at the top and the positions of residues mutated are indicated by asterisks. (B) The morphology of MCF-10A cells expressing GFP is shown in the left panels and of cells expressing Vav1Y3F is shown in the right panels. Note that cells expressing Vav1Y3F are flatter and more spread and have more ruffles and lamellipodia than the cells expressing GFP. Data are representative of more than 10 independent experiments. (C) MCF-10A cells expressing GFP and Vav1Y3F were seeded in transwell chambers and allowed to migrate overnight towards assay media without or with 20 ng/ml EGF. The average number of cells migrated in five 20x fields per transwell was determined. Migration is expressed as the percentage of migration of Vav1Y3F cells in media without EGF (set to 100%) for each individual experiment. Data are the average plus standard deviation of 8 independent experiments.

To examine the effects of activated Vav in MCF-10A mammary epithelial cells, we constructed a retroviral vector encoding an activated form of Vav1, referred to as Vav1Y3F, that contains phenylalanine substitutions for three acidic domain tyrosine residues (Y142, Y160 and Y174) (Fig. 1A). These tyrosine residues are able to participate in autoinhibitory interactions with the DH domain of Vav1. Phosphorylation prevents the interaction and leads to activation of Vav1 GEF activity [25,27]. In addition, mutation of these residues to phenylalanine has been shown to result in a Vav1 protein with constitutive activity [19,20].

The activated Vav1Y3F variant was expressed in MCF-10A cells, a line of immortalized, non-transformed human mammary epithelial cells, because they display a non-motile phenotype in the absence of growth factors. MCF-10A cells were infected with retroviral vectors encoding either GFP or Vav1Y3F-GFP, and the morphology of infected cells was compared. Expression of the GFP-tagged form of Vav1Y3F caused a change in the morphology of MCF-10A cells that was not observed in cells expressing GFP alone. The GFP-expressing cells displayed a cobblestone appearance indistinguishable from non-infected MCF-10A cells. In contrast, cells expressing Vav1Y3F were flatter and more spread and displayed more ruffles and lamellipodia (Fig. 1B).

Because Vav is a GEF for Rac, Rho, and Cdc42, and these GTPases play important roles in migration [49], we examined the effect of Vav1Y3F expression on migration. MCF-10A cells require EGF stimulation to migrate; however, the expression of certain proteins such as H-Ras causes the cells to migrate in the absence of EGF [50]. The ability of cells expressing GFP and GFP-tagged Vav1Y3F to migrate was examined using a transwell assay. In the absence of EGF, GFP-expressing cells do not migrate. However, upon EGF stimulation, the migration of these cells increases 80- to 100-fold. Expression of Vav1Y3F caused an 80- to 100- fold stimulation of MCF-10A cell migration relative to expression of GFP alone. In addition, Vav1Y3F-GFP enhanced migration in the presence of EGF (Fig. 1C).

### Function-blocking mutations in the DH, PH, or CR domains suppress Vav1Y3F activities

To determine which domains of Vav1 are required for the morphological changes and increased migration of MCF-10A cells, variant forms of Vav1Y3F containing inactivating point mutations in various domains were expressed in MCF-10A cells (Fig. 1A). It has previously been shown that in addition to the catalytic DH domain, the CR domain is required for the GEF activity of Vav1, Vav2, and Vav3 in vitro [6,20,21]. In contrast, inactivation of the PH domain of Vav isoforms has no effect on exchange activity in vitro but inhibits Vav activity in cells by an unknown mechanism [20-22]. We examined the effects of similar inactivating mutations in these domains on the ability of Vav1Y3F to stimulate morphological changes and increase migration in MCF-10A cells. The Vav1Y3F/DH* protein contains a L213Q mutation which had previously been found to inactivate the GEF function of this domain [20,32,51]. The Vav1Y3F/PH* protein contains a leucine substitution for tryptophan residue 495 that is conserved in nearly all PH domains. This tryptophan contributes a side chain to the hydrophobic core of PH domains and is thought to have a role in domain stability [52]. The Vav1Y3F/CR* mutant contains a serine substitution for cysteine 529 which contributes to formation of one of the zinc finger motifs in the CR domain. The latter two mutations have been shown to inactivate the transforming ability of oncogenic or active forms of Vav1 in NIH3T3 cells [20,22,53]. In addition, the C529S mutation blocks the guanine nucleotide exchange activity of Vav3 in vitro [6] and of Vav1 in nucleotide loading of Rac1 in vitro and in cells [20,32]. All three of these mutated proteins were also GFP tagged at their C-termini.

**Figure 2:**
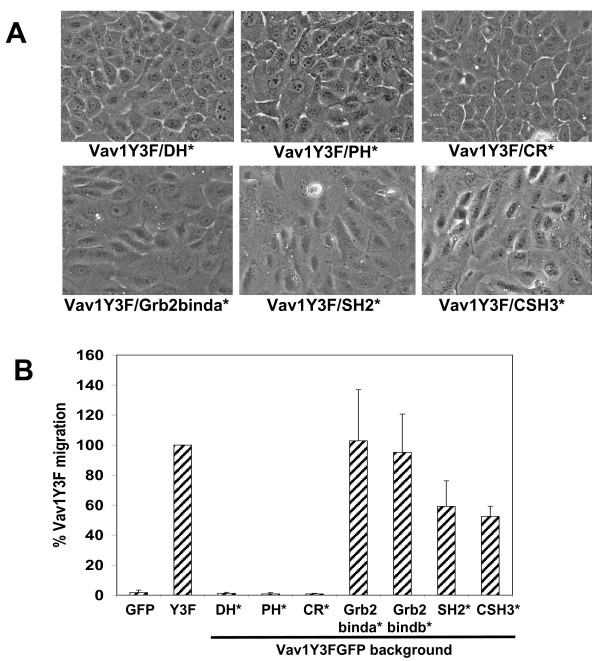
**Inactivation of different Vav1Y3F domains reveals roles for GEF and scaffolding activities in Vav1Y3F-induced phenotypes**. (A) Morphology of MCF-10A cells expressing forms of Vav1Y3F with inactivating mutations in different structural domains. Data are representative of 3 or more independent experiments. (B) Cumulative data from experiments showing migration of GFP and different forms of Vav1Y3F through transwells in the absence of EGF. GFP and Vav1Y3F were included in each experiment and migration is expressed as the percentage of migration of Vav1Y3F (set to 100%) for each individual experiment. Data are the average plus standard deviation of two (PH* and CR*) to five (SH2* and SH3*) independent experiments.

The appearance of MCF-10A cells expressing the Vav1Y3F/DH*, Vav1Y3F/PH*, and Vav1Y3F/CR* proteins were indistinguishable from the GFP expressing cells, indicating that mutation of these domains prevents Vav1Y3F induced morphological alterations (Fig. 2A, top panels). In addition, cells expressing these proteins did not migrate in the absence of EGF (Fig. 2B) and did not stimulate increased migration over that of GFP-expressing cells in the presence of EGF (data not shown). These data suggest that the DH, PH, and CR domains of Vav1 are required for its ability to cause cell spreading, ruffle formation, and increased migration.

### Mutations in the adaptor region of Vav1Y3F have variable effects on cell morphology and migration

Vav1 is known to interact with many different proteins through its C-terminal adaptor region [14]. To investigate whether these interactions are required for the migratory phenotype caused by Vav1Y3F expression, we generated mutants that disrupted known interactions of the adaptor region. The interaction between the N-SH3 domain of Vav1Y3F and the C-terminal SH3 domain of Grb2 was inhibited by substitutions of tyrosine for tryptophan residue 637 (W637Y) and alanine for proline at residue 657 (P657A). These two residues are in the interface between Vav1 and Grb2, and substitutions at these sites were found to decrease the binding affinity between the two proteins 40- and 9- fold, respectively [47]. These proteins were termed Vav1Y3F/Grb2binda* and Vav1Y3F/Grb2bindb*. To disrupt the ability of the SH2 domain of Vav1Y3F to bind phosphotyrosine, Vav1Y3F/SH2* was generated by mutating arginine at residue 696 in the active site to lysine (Fig. 1A). This arginine is in the FLVR motif required for binding to phosphotyrosines [54,55]. The N-SH3 and C-SH3 domains were inactivated by mutating P651 and P833 to leucines to make Vav1Y3F/NSH3* and Vav1Y3F/CSH3*. Mutation of P833 to leucine in Vav1 blocks interaction with polyproline sequences [40,56,57], and the equivalent SH3 domain mutation in Sem5 blocks its function in C. elegans [58,59]. The locations of these mutations in Vav1 are illustrated in figure 1A.

None of the mutations in the adaptor region of Vav1Y3F affected its ability to induce a flattened, well-spread morphology (Fig. 2A, bottom panels, and data not shown). In addition, none of the mutations in the N-SH3 domain had an effect on the migratory activity of Vav1Y3F (Fig. 2B, data not shown). However, mutation of the SH2 or the C-SH3 domains suppressed the strong migratory phenotype of Vav1Y3F. Vav1Y3F/SH2* and Vav1Y3F/CSH3* expressing cells showed 2-fold lower migration vs. Vav1Y3F in the absence of EGF (Fig. 2B). Therefore, the SH2 and C-SH3 domains appear to be required for maximal Vav1Y3F stimulated migration.

### MCF-10A cells expressing Vav1Y3F have increased basal activation of Rac1, Pak, and ERK1/2, and inactivation of different domains has varying effects on the activity state of these signaling molecules

**Figure 3:**
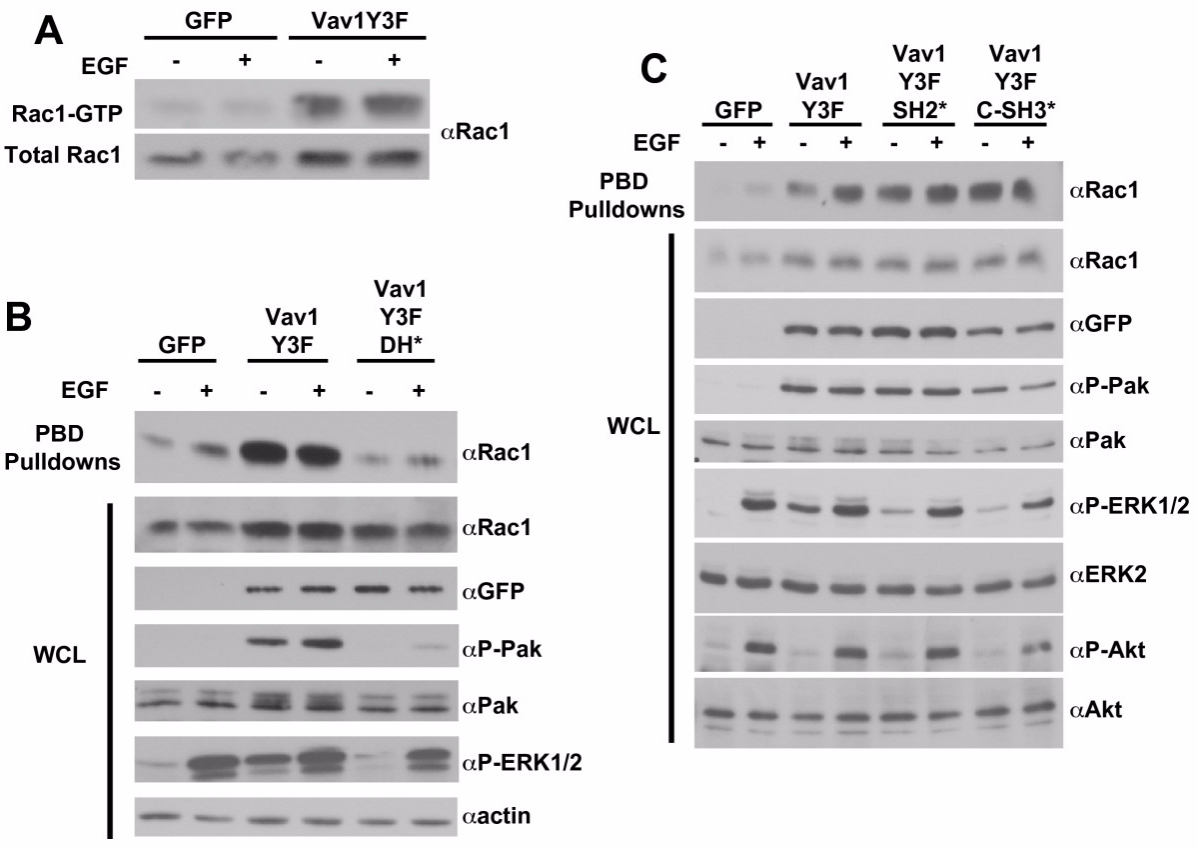
**Different Vav1Y3F domains are required to increase levels of Rac1-GTP and phosphorylated Pak and ERK1/2**. (A) After overnight starvation, MCF-10A cells expressing GFP or Vav1Y3F were left unstimulated or stimulated with EGF. PBD pulldown assays were performed as described in the Materials and Methods. Levels of Rac1-GTP and total Rac1 are shown as indicated. Data are representative of 4 independent experiments. (B) MCF-10A cells expressing GFP, Vav1Y3F, and Vav1Y3F/DH* were treated as in panel A and PBD assays were performed. In addition, whole cell lysate (WCL) samples were blotted for Vav1Y3FGFP, phosphorylated Pak, total Pak, phosphorylated ERK1/2, and actin as indicated. Data are representative of 2 independent experiments. (C) Levels of Rac1-GTP, total Rac1, Vav1Y3FGFP, and phosphorylated and total Pak, ERK1/2, and Akt in lysates from MCF-10A cells expressing GFP, Vav1Y3F, Vav1Y3F/SH2*, or Vav1Y3F/C-SH3* were determined as in panel B. Data are representative of 3 independent experiments.

Because Vav1 is known to activate Rho GTPases and aberrant activation of Rho GTPases can result in increased migratory and invasive phenotypes, the levels of Rac1-GTP and Cdc42-GTP in MCF-10A cells expressing GFP, Vav1Y3F, or Vav1Y3F/DH* proteins were measured using a p21 binding domain (PBD) pulldown assay. Expression of Vav1Y3F in MCF-10A cells resulted in high levels of Rac1-GTP in unstimulated cells, and the Rac1-GTP levels did not increase after EGF stimulation of the cells (Fig. 3A). Lysates from GFP and Vav1Y3F/DH* expressing cells contained little Rac1-GTP in the absence or presence of EGF stimulation (Fig. 3A and 3B). Although Vav1Y3F expression caused robust activation of Rac1 in MCF-10A cells, it did not increase levels of Cdc42-GTP (data not shown).

Vav1Y3F expressing MCF-10A cells also contained increased basal phosphorylated Pak and phosphorylated ERK1/2 as well as higher levels of phosphorylated Pak following EGF stimulation (Fig. 3B and 3C). However, cells expressing Vav1Y3F did not exhibit enhanced phosphorylation of Akt either in the absence or presence of EGF stimulation (Fig. 3C). Vav1Y3F proteins containing mutations that inactivated the DH, PH, and CR domains did not increase levels of Rac1-GTP, phosphorylated Pak or phosphorylated ERK1/2 (Fig. 3B and data not shown). In contrast, mutation of the SH2 and C-SH3 domains of Vav1Y3F did not reduce the induction of Rac1-GTP or phosphorylated Pak, but did reduce the level of ERK phosphorylation two-fold (Fig. 3C). These results suggest that the ability of Vav1Y3F to activate Rac1 through its DH, PH, and CR domains contributes to its phenotypic effects in MCF-10A cells. In addition, the induction of phosphorylated ERK1/2 in the absence of EGF stimulation correlates with the migratory activity of Vav1Y3F as evidenced by the effects of the SH2 and C-SH3 mutations on migration and phosphorylated ERK1/2.

### Expression of Vav1Y3F in MCF-10A cells causes secretion of an EGF receptor ligand that stimulates migration

**Figure 4:**
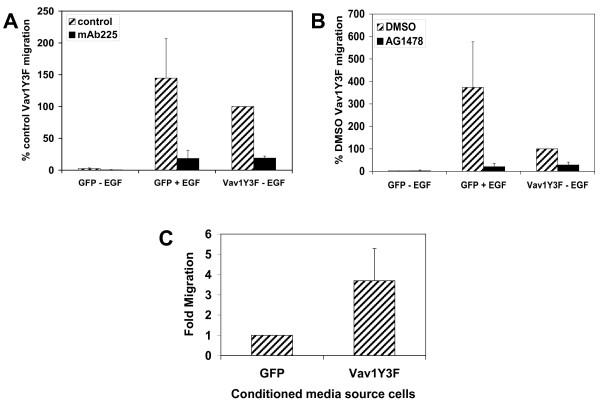
**Vav1Y3F increases migration of MCF-10A cells by causing secretion of an EGF receptor-dependent factor**. (A) MCF-10A cells expressing GFP or Vav1Y3F were lifted and pretreated with hybridoma medium (control) or with mAb225 for 30 minutes at 37°C. Cells were then seeded in transwells in wells containing assay media minus and plus 5 ng/ml EGF for GFP or assay media minus EGF for Vav1Y3F and allowed to migrate overnight. The data are graphed as the percentage of migration of control Vav1Y3F cells in media without EGF (set to 100%) for each individual experiment. Data are the average plus standard deviation for 3 transwells. (B) MCF-10A cells expressing GFP or Vav1Y3F were lifted and pretreated with DMSO (control) or 300 nM AG1478 for 30 minutes at 37°C. Cells were then seeded in transwells in wells containing assay media plus DMSO -/+ EGF or assay media plus AG1478 -/+ EGF as indicated and allowed to migrate overnight. Data are expressed as in panel A and are the average plus standard deviation for 3 transwells. (C) Conditioned media was collected from MCF-10A cells expressing GFP or Vav1Y3F as described in the Materials and Methods. Transwells were placed in wells containing the different conditioned medias and naïve MCF-10A cells were seeded in the top of the transwell. Cells were allowed to migrate overnight and migration was analyzed as in panel A. Data are expressed as the fold migration of cells with migration of cells in GFP cell conditioned media set to 1. Data are the average plus standard deviation of 5 independent experiments.

It has been shown previously that activation of Raf in MCF-10A cells causes secretion of EGF receptor ligands [60]. In addition, co-activation of ErbB2 and the TGF-β receptor in MCF-10As causes secretion of EGF receptor dependent factors that stimulate migration [61]. To determine whether the migration of Vav1Y3F-expressing MCF-10A cells is dependent on the secretion of an EGF receptor ligand, the migration assay was performed in the presence of the EGF receptor inhibitory antibody, mAb225. This antibody binds to the extracellular domain of the EGF receptor and blocks ligand binding, resulting in the inhibition of EGF receptor signaling [62]. Both the migration of GFP expressing cells in the presence of EGF and migration of Vav1Y3F expressing cells in the absence of EGF were blocked by mAb225 (Fig. 4A).

The migration assay was also performed in the presence of AG1478, a small molecule inhibitor of the EGF receptor kinase domain, to investigate whether EGF receptor kinase activity is required for the increased migratory ability of Vav1Y3F expressing MCF-10A cells. AG1478 inhibited migration of GFP control cells stimulated with EGF as well as EGF-independent migration of Vav1Y3F cells (Fig. 4B). These results indicate that Vav1Y3F-induced MCF-10A migration requires ligand binding and kinase activity of the EGF receptor.

If Vav1Y3F stimulates the secretion of an EGF receptor ligand, conditioned medium collected from MCF-10A cells expressing Vav1Y3F may cause migration of uninfected MCF-10A cells. To examine this possibility, we examined the ability of conditioned medium from these cells to induce migration of uninfected MCF-10A cells. GFP or Vav1Y3F expressing cells were cultured for 48 hours in medium lacking EGF. The resulting conditioned medium from GFP expressing cells did not enhance migration of the naïve MCF-10A cells (Fig. 4C). In contrast, conditioned medium from Vav1Y3F expressing cells induced a 4-fold increase in migration of the MCF-10A cells (Fig. 4C). These data strongly suggest that expression of Vav1Y3F in MCF-10A cells results in secretion of an EGF receptor ligand that stimulates migration by activating the EGF receptor. However, we cannot rule out that the presence of EGF receptor-independent factors in the Vav1Y3F conditioned media are responsible for migration of the naïve MCF-10A cells.

### Vav1Y3F increases basal ERK1/2 phosphorylation in a manner dependent on EGF receptor activation

**Figure 5:**
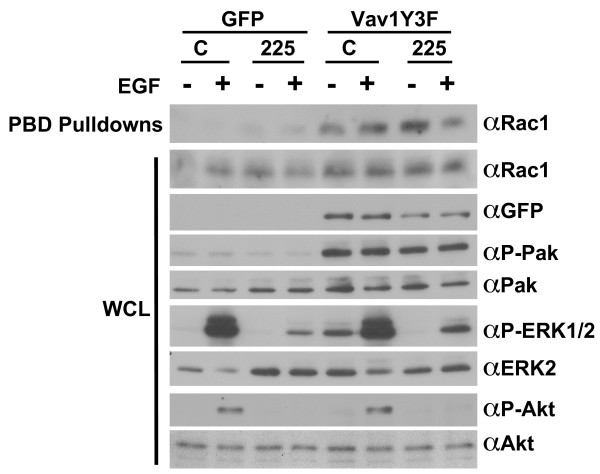
**Increased basal phosphorylated ERK1/2 stimulated by Vav1Y3F is blocked by mAb225 pretreatment of the cells**. MCF-10A cells expressing GFP or Vav1Y3F were starved in assay media containing hybridoma media as a control (C) or mAb225 overnight. The cells were then left unstimulated or stimulated with 5 ng/ml EGF for 5 minutes. Cells were lysed and PBD pulldown assays were performed. WCL samples were also blotted for levels of total Rac1, Vav1Y3FGFP, phosphorylated Pak, total Pak, phosphorylated ERK1/2, total ERK2, phosphorylated Akt, and total Akt as indicated. Data are representative of 3 independent experiments.

Rac1 is activated and Pak and ERK1/2 are phosphorylated following both Vav1 and EGF receptor activation [1,11,14,63-65]. To investigate whether Vav1 stimulation of these pathways is dependent on ligand binding to the EGF receptor, we starved cells expressing GFP or Vav1Y3F in the presence of mAb225, stimulated half the samples, and then examined the levels of Rac1-GTP and Pak and ERK1/2 phosphorylation in the cell lysates. Treatment of Vav1Y3F cells with mAb225 did not diminish the stimulation of Rac1-GTP levels or Pak phosphorylation induced by Vav1Y3F, however mAb225 eliminated Vav1Y3F-induced constitutive phosphorylation of ERK1/2 (Fig. 5). In addition, inhibition of MEK, the upstream activator of ERK, with U0126 blocked both the EGF-dependent migration of GFP control cells and the EGF-independent migration of Vav1Y3F cells (see additional file 1). These results indicate that Vav1Y3F activates the ERK pathway indirectly through autocrine stimulation of the EGF receptor and that ERK activation downstream of EGF receptor stimulation is required for the increased MCF10A migration resulting from Vav1Y3F expression.

Based on the results in this report, we propose the following mechanism for Vav1Y3F stimulation of MCF-10A cell migration. Expression of Vav1Y3F causes activation of one or more Rho GTPases leading to production of a secreted factor that stimulates migration through binding to the EGFR. The GEF activity of Vav is required for secretion of the autocrine factor and migration because Y3F/DH*, Y3F/PH*, and Y3F/CR* do not stimulate migration. While the Rho family GTPase responsible for secretion of the EGF receptor ligand was not identified, Rac1 represents one candidate family member since GTP loading of Rac was strongly stimulated by Vav1Y3F and this stimulation as well as phosphorylation of a downstream target of Rac, Pak, was independent of EGF receptor ligand binding. Rac and/or other Vav1-activated Rho family GTPases may collaborate with EGFR signaling to stimulate cell migration since the level of MCF-10A cell migration stimulated by Vav1Y3F-conditioned medium is not as strong as that observed in Vav1Y3F expressing cells. This collaboration could involve Vav1Y3F enhancement of EGF stimulated pathways since Vav1 binds to activated EGFR.

The results from this study also implicate the Vav SH2 and C-SH3 domains in Vav1Y3F-stimulated migration because Y3F/SH2* and Y3F/SH3* were only half as effective as Vav1Y3F in inducing migratory activity. These mutants stimulate Rac1 and Pak activation to the same level as Vav1Y3F but activate ERK half as well, indicating that the activation of ERK correlates with the migration stimulating activity of the Vav SH3 and SH2 domain mutants. One possibility is that the SH2 and C-SH3 domains recruit a factor that cooperates with Rac1 to stimulate production of the autocrine factor. The Vav1 SH2 domain was also found by del Pozo et al. to be required for cooperation with V12Rac in the induction of T cell spreading. Although the DH domain was required for spreading of T cells overexpressing Vav1 alone, Vav1DH* could still synergize with V12Rac in inducing cell spreading while Vav1 containing an SH2 mutation could not [23]. Thus, Vav has functions that are both dependent and independent of its ability to activate Rho GTPases.

Previous studies provided evidence that Vav is critically involved in receptor pathways that couple to ERK [66-69]. For example, Tybulewicz and colleagues found that ERK activation is impaired downstream of T cell receptor (TCR) activation in Vav1-/- CD4^+ ^T cells [66]. In subsequent studies, they showed that Vav1 appears to activate ERK downstream of TCR activation through a pathway involving LAT phosphorylation and Sos activation as well as phospholipase C activation and membrane recruitment of RasGRP1 [67]. In addition, knock down of endogenous Vav protein in the cultured *Drosophila *S2 cells overexpressing DER, the *Drosophila *homolog of the EGF receptor, blocked ERK phosphorylation following stimulation of DER, suggesting that Vav is required for phosphorylation of ERK downstream of DER [68]. Data presented here suggest that Vav1 can also activate ERK in MCF-10A cells through an indirect pathway involving secretion of an EGF receptor ligand. Differences in the signaling pathways that couple activated Vav to ERK in different cell types and through different ligands are likely due to cell type specific expression of different signaling proteins. For example, breast and other epithelial cells lack LAT and other proteins involved in ERK activation following TCR stimulation.

While Vav1 expression is normally restricted to hematopoietic cells, it has been shown to be expressed in neuroblastoma and gastric epithelial tumor cells and Vav2 and Vav3 are overexpressed in a variety of tumor cells [7-10]. We have preliminary data showing that expression of active forms of Vav2 also exhibit increased migration of MCF-10A cells in the absence of EGF (Moores and Brugge, unpublished results). Therefore, it is possible that Vav proteins could contribute to the activation of Rac and ERK pathways during tumor progression, possible leading to changes in the migratory behavior of tumor cells.

## Conclusion

Expression of Vav1Y3F in MCF-10A mammary epithelial cells causes an increase in migration of the cells in the absence and presence of exogenous EGF. The increased migration of Vav1Y3F expressing cells is dependent on secretion of an autocrine EGF receptor ligand, and maximal migration requires functional DH, PH, CR, SH2 and C-SH3 domains. Activation of ERK downstream of Vav1 is dependent on autocrine EGF receptor stimulation while Vav1Y3F stimulates Rac1 and PAK activation independent of the EGF receptor. Secretion of an autocrine ligand is a novel mechanism by which Vav isoforms may activate the MAP kinase pathway in non-hematopoietic cells.

## Methods

### Reagents and cell culture

Culture of MCF-10A cells was described in [70]. MCF-10A cells expressing the ecotropic receptor were made by retroviral transfection of low passage MCF-10A cells with a mEcoRneo retrovirus, followed by selection with neomycin. Antibodies used included mouse anti-phosphotyrosine 4G10 (T. Roberts, Dana Farber Cancer Institute, Boston, MA), mouse anti-Rac1 and mouse anti-Cdc42 (BD-Transduction Labs, San Diego, CA), rabbit anti-pS198/S203 PAKα (M. Greenberg, Children's Hospital, Boston, MA), rabbit anti-phosphoERK1/2 (Biosource, Camarillo, CA) mouse anti-GFP, rabbit anti-Pak, and rabbit anti-ERK2 (Santa Cruz Biotechnology, Inc., Santa Cruz, CA), rabbit anti-phosphoAkt S473 (Cell Signaling Technology, Inc., Beverly, MA), and rabbit ant-Akt 1199 described in [71]. AG1478 and U0126 were purchased from Calbiochem (San Diego, CA). Monoclonal antibody 225 was obtained from the lab of D. Lauffenburger (Massachusetts Institute of Technology, Boston, MA) or produced from the HB-8508 hybridoma obtained from American Type Culture Collection (Manassas, VA). The pEQPAM3(-E) and pEQEnvE plasmids were kindly provided by M. Roussel (St. Jude Children's Research Hospital, Memphis, TN).

### Generation of Vav1Y3F expression plasmids

Mutations of the tyrosines to phenylalanine in the acidic domain of Vav1 in the pCF1.HA plasmid were generated using the QuikChange kit (Stratagene, La Jolla, CA). The Gateway cloning system (Invitrogen, Carlsbad, CA) was used to subclone Vav1Y3F into pMXuGFP (kindly provided by E. Koh, Whitehead Institute, Cambridge, MA), resulting in a retroviral vector encoding Vav1Y3F with a C-terminal GFP tag. Mutations in the different domains of Vav1Y3F in pMXuGFP were made using the QuikChange kit (Stratagene, La Jolla, CA).

### Production of retrovirus encoding GFP and Vav1Y3F proteins and infection of MCF-10A cells

293T cells were co-transfected with vectors encoding gag/pol, ecotropic envelope, and Vav1Y3F proteins (pEQPAM3(-E), pEQEnvE, and pMXVav1Y3FuGFP, respectively) using the calcium phosphate method. Virus was collected at 48 hours after transfection, 0.45 μm filtered, aliquoted, and frozen at -80°C. MCF-10A cells expressing the ecotropic receptor were infected with GFP or wild-type or mutated Vav1Y3F viruses and used 48 hours later for migration or biochemistry experiments.

### Transwell migration assays

Transwell assays and conditioned media production were performed as described in Seton-Rogers et al. [61], except cells were not starved before lifting them and seeding them in the transwells, and conditioned media was collected after 48 hours.

### Preparation of monoclonal antibody 225

Media containing monoclonal antibody 225 was harvested from hybridoma cells and filtered through a 0.2 μm filter. The media was concentrated and an ammonium sulfate precipitation was performed to isolate the monoclonal antibody. The pellet was dissolved in phosphate buffered saline and the antibody solution was dialyzed into phosphate buffered saline to remove ammonium sulfate. The activity of the resulting antibody solution was determined by measuring its effect on EGF stimulated migration and EGF receptor phosphorylation in MCF-10A cells. The amount of the antibody solution used in migration and PBD assays had activity equivalent to that seen with 10 μg/ml of purified mAb225 obtained from the lab of D. Lauffenburger (Massachusetts Institute of Technology, Boston, MA).

### PBD pulldown assays and immunoblotting

MCF-10A cells infected in 6 well plates with Vav1Y3F retroviruses were starved in assay media (DMEM/F12, 2% horse serum, 0.5 μg/ml hydrocortisone, 100 ng/ml cholera toxin, 10 μg/ml insulin, and 1x penicillin/streptomycin) overnight starting at 36 hours after infection. The next morning, the cells were left unstimulated or stimulated with 20 ng/ml EGF for 5 minutes, washed with PBS, and lysed in PBD lysis buffer (50 mM Tris, pH 7.6, 150 mM NaCl, 1% Triton X-100, 0.5 mM MgCl_2_, 1 mM NaF, 1 mM β-glycerophosphate, 1 mM Na_3_VO_4_, 100 μg/ml PMSF, 10 μg/ml leupeptin, and 2 μg/ml aprotinin) containing 10 μg of GST-PBD per sample. Lysates were clarified at 13,000 rpm for 5 minutes at 4°C. Small aliquots of lysates were combined with 2x SDS sample buffer for whole cell lysate samples and the rest was incubated with 30 μl/sample of a 1:1 slurry of glutathione agarose beads in PBD lysis buffer on a rotator at 4°C for 45 minutes. Beads were washed and 2x SDS sample buffer was added to each sample. Immunoblotting was performed as described in Seton-Rogers et al [61].

## Competing interests

The authors declare that they have no competing interests.

## Authors' contributions

JLW conceived the study, designed and performed all the experiments, interpreted the results, and wrote the manuscript. JSB and SLM participated in the conception and design of the study as well as interpretation and discussion of results.

## Supplementary Material

Additional File 1 **Increased migration stimulated by Vav1Y3F expression is blocked by U0126**. MCF-10A cells expressing GFP or Vav1Y3F were lifted and pretreated with DMSO (control) or 5 μM U0126 for 30–60 minutes at 37°C. Cells were then seeded in transwells in wells containing assay media plus DMSO -/+ EGF or assay media plus U0126 -/+ EGF as indicated and allowed to migrate overnight. Data are expressed as the percentage of migration of control Vav1Y3F cells in media without EGF (set to 100%) for each individual experiment and are the average plus standard deviation for 3 transwells. Click here for file
