# Supplementary figures and images for: An active form of Vav1 induces migration of mammary epithelial cells by stimulating secretion of an epidermal growth factor receptor ligand
Source: Cell Commun Signal. 2006 May 18;4:5. doi: 10.1186/1478-811X-4-5 (PMC1524963; doi:10.1186/1478-811X-4-5)

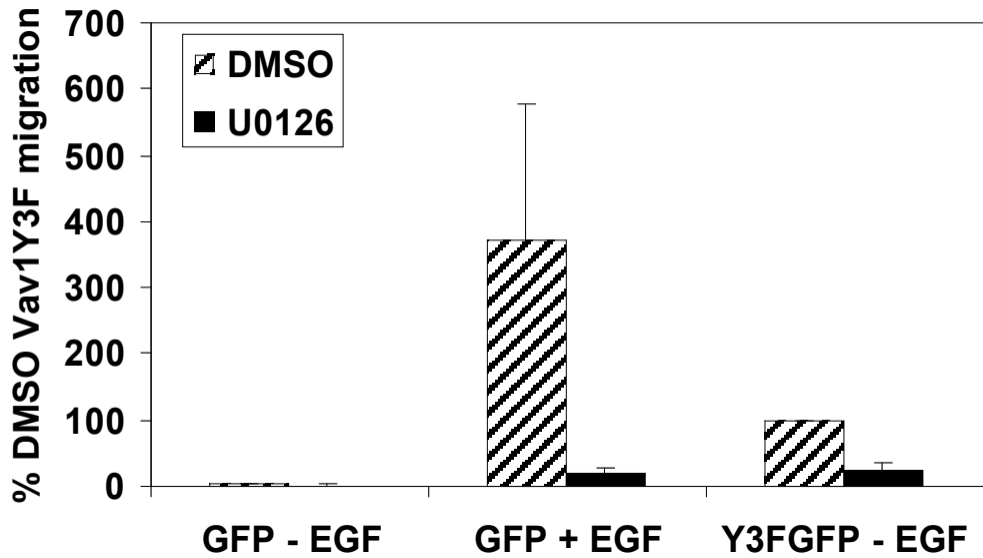

Supplement: Additional File 1 — Increased migration stimulated by Vav1Y3F expression is blocked by U0126. MCF-10A cells expressing GFP or Vav1Y3F were lifted and pretreated with DMSO (control) or 5 μM U0126 for 30–60 minutes at 37°C. Cells were then seeded in transwells in wells containing assay media plus DMSO -/+ EGF or assay media plus U0126 -/+ EGF as indicated and allowed to migrate overnight. Data are expressed as the percentage of migration of control Vav1Y3F cells in media without EGF (set to 100%) for each individual experiment and are the average plus standard deviation for 3 transwells. [file 1478-811X-4-5-S1.pdf]
